# Exploring the molecular basis of resistance to *Botrytis cinerea* in chickpea genotypes through biochemical and morphological markers

**DOI:** 10.7717/peerj.15560

**Published:** 2023-06-20

**Authors:** Richa Thakur, Sucheta Sharma, Rajni Devi, Asmita Sirari, Rahul Kumar Tiwari, Milan Kumar Lal, Ravinder Kumar

**Affiliations:** 1 Punjab Agricultural University, Ludhiana, Punjab, India; 2 ICAR-Central Potato Research Institute, Shimla, India

**Keywords:** Chickpea, Antioxidant, Innoculation, *Botrytis cinerea*, Isozymes

## Abstract

Chickpea (*Cicer arietinum* L.) is an important pulse crop around the globe and a valuable source of protein in the human diet. However, it is highly susceptible to various plant pathogens such as fungi, bacteria, and viruses, which can cause significant damage from the seedling phase until harvest, leading to reduced yields and affecting its production. *Botrytis cinerea* can cause significant damage to chickpea crops, especially under high humidity and moisture conditions. This fungus can cause grey mould disease, which can lead to wilting, stem and pod rot, and reduced yields. Chickpea plants have developed specific barriers to counteract the harmful effects of this fungus. These barriers include biochemical and structural defences. In this study, the defence responses against *B. cinerea* were measured by the quantification of biochemical metabolites such as antioxidant enzymes, malondialdehyde (MDA), proline, glutathione (GSH), H_2_O_2_, ascorbic acid (AA) and total phenol in the leaf samples of chickpea genotypes (one accession of wild *Cicer* species, *viz*. *Cicer pinnatifidum*188 identified with high level of resistance to Botrytis grey mould (BGM) and a cultivar, *Cicer arietinum*PBG5 susceptible to BGM grown in the greenhouse). Seedlings of both the genotypes were inoculated with (1 × 10^4^ spore mL^−1^) inoculum of isolate 24, race 510 of *B. cinerea* and samples were collected after 1, 3, 5, and 7 days post-inoculation (dpi). The enhanced enzymatic activity was observed in the pathogen-inoculated leaf samples as compared to uninoculated (healthy control). Among inoculated genotypes, the resistant one exhibited a significant change in enzymatic activity, total phenolic content, MDA, proline, GSH, H_2_O_2_, and AA, compared to the susceptible genotype. The study also examined the isozyme pattern of antioxidant enzymes at various stages of *B. cinerea* inoculation. Results from scanning electron microscopy (SEM) and Fourier transform infrared (FTIR) spectroscopy revealed that BGM had a more significant impact on susceptible genotypes compared to resistant ones when compared to the control (un-inoculated). In addition, SEM and FTIR spectroscopy analyses confirmed the greater severity of BGM on susceptible genotypes compared to their resistant counterparts. Our results suggest the role of antioxidant enzymes and other metabolites as defence tools and biochemical markers to understand compatible and non-compatible plant-pathogen interactions better. The present investigation will assist future plant breeding programs aimed at developing resistant varieties.

## Introduction

Chickpea (*Cicer arietinum* L.) is an important leguminous crop and a valuable source of protein in the human diet. The beneficial effects of chickpeas on both soil and human health have been well recognized (Merga & Haji, 2019). Chickpea is cultivated on 11.12 million hectares with a production of 11.6 million tons (Food and Agriculture Organization of the United Nations (FAO), 2020). However, it is highly susceptible to various plant pathogens such as fungi, bacteria, and viruses, which can cause significant damage from the seedling phase until harvest, leading to reduced yields and affecting its production. The most common fungal pathogens affecting chickpeas are *Fusarium oxysporum, Ascochyta rabiei, Botrytis cinerea, Rhizoctonia solani*, and *Sclerotinia sclerotiorum*, while bacterial diseases include bacterial blight caused by *Xanthomonas axonopodis pv. ciceri* and bacterial wilt caused by *Ralstonia solanacearum*. Viral diseases affecting chickpeas include *Chickpea chlorotic dwarf virus*, *Chickpea stunt disease*, and *Chickpea leafroll virus*. Managing these pathogens in chickpea cultivation requires an integrated approach that includes the use of disease-resistant varieties, crop rotation, seed treatment, cultural practices, and chemical control measures.

Botrytis grey mould (BGM) is one of the chickpea’s important and destructive fungal diseases. It is caused by a necrotrophic fungus *B. cinerea* Pers. ex. Fr. The fungus infects all the aerial parts of chickpea plants throughout the growth stages with an annual yield loss of 50% or more (Youssef & Roberto, 2014, Thakur et al., 2023). The fungus presents morphologically as dense white colonies on the twig stem, and leaves, which mature into water-soaked lesions. As a necrotrophic pathogen, the disease persists as sclerotia on plant detritus and soil (Kumar et al., 2008, Pandey, Kumar & Mishra, 2010). Controlling BGM in agricultural settings is challenging due to its various mechanisms of infection, diverse host range, and ability to survive in different forms (Brandhoff et al., 2017). Although the repeated use of fungicides can prevent BGM from becoming endemic, it is not a cost-effective technique for low-income farmers and could potentially lead to the development of resistant strains of the fungus (Zouari et al., 2016). Previously it has been reported that wild *Cicer* species were a valuable source of resistance to BGM (Haware, Narayana Rao & Pundir, 1992). Therefore, it was of higher importance to consolidating available resistance sources with other management alternatives to diminish the effect of the disease.

During the plant-pathogen interactions, various fungi can affect host plants in different ways, which include the detrimental effects of released toxins, absorption of host nutrients and suppression of plant defence by silencing certain genes and induced programmed cell death (Mbengue et al., 2016; Nalcaci et al., 2021; Xu et al., 2021). Plants have developed specific defenses to counteract the harmful effects of fungi. These defenses include biochemical barriers such as hydrolytic enzymes, phytoalexins, fungal toxins, and structural barriers such as lignin and hydroxyproline-rich cell wall proteins. These mechanisms work together to prevent or limit fungal infection and protect the plant from damage (Boots & Best, 2018; Kumar et al., 2021; Pandey & Basandrai, 2021). Oxidative burst involving Reactive oxygen species (ROS) released by host plants is one of the earlier detectable responses during the pathogenic fungus attack (Li et al., 2016; Smirnoff & Arnaud, 2019). ROS may act as a second messenger for activating protective proteins and defense signaling (Mittler et al., 2004; Das & Roychoudhury, 2014), which can render resistance against pathogens (Bellincampi, Cervone & Lionetti, 2014). Many necrotrophic pathogens have been reported to use oxidative forces to attack, invade and destroy plant tissues. ROS’s role in cell death induction by *B. cinerea* has been suggested (Qi et al., 2017; Li et al., 2018). Plants have developed antioxidant defence systems to avoid oxidative damage from ROS, including enzymatic and non-enzymatic systems, which co-evolved with aerobic metabolism (Khaledi, Taheri & Falahati-Rastegar, 2017). These protective enzymes include superoxide dismutase (SOD), catalase (CAT), ascorbate peroxidase (APX), guaiacol peroxidase (GPX), polyphenol oxidase (PPO), and phenylalanine ammonia-lyase (PAL). In contrast, several molecules, such as glutathione, proline, ascorbate (AA), phenolic and carotenoids provide non-enzymatic protection (Kasote et al., 2015). The overall balance between the amount of ROS produced and the components of the antioxidant defense system may decide a plant’s susceptibility to oxidative stresses.

A huge chickpea crop loss in the Gurdaspur region of Punjab, India, occurred in the last 5 years due to BGM infection. The resistance sources are limited, and management mostly relies on chemical formulations. The information on the interaction of *B. cinerea* with chickpea and parameters associated with oxidative stress is elusive. Also, the biochemical studies on chickpea genotypes during *B. cinerea* infections have not been explored yet. In the present study, we attempted to investigate the changes in biochemical molecules (enzymatic and non-enzymatic) associated with defence mechanisms induced during BGM infection in resistant (an accession, *Cicer pinnatifidum*188 of wild *Cicer* species) and susceptible (a cultivar PBG5 of *Cicer arietinum*) chickpea genotypes. The study will assist in future plant breeding programs to develop resistant genotypes.

## Materials and Methods

### Plant material and growth conditions

**Figure 1:**
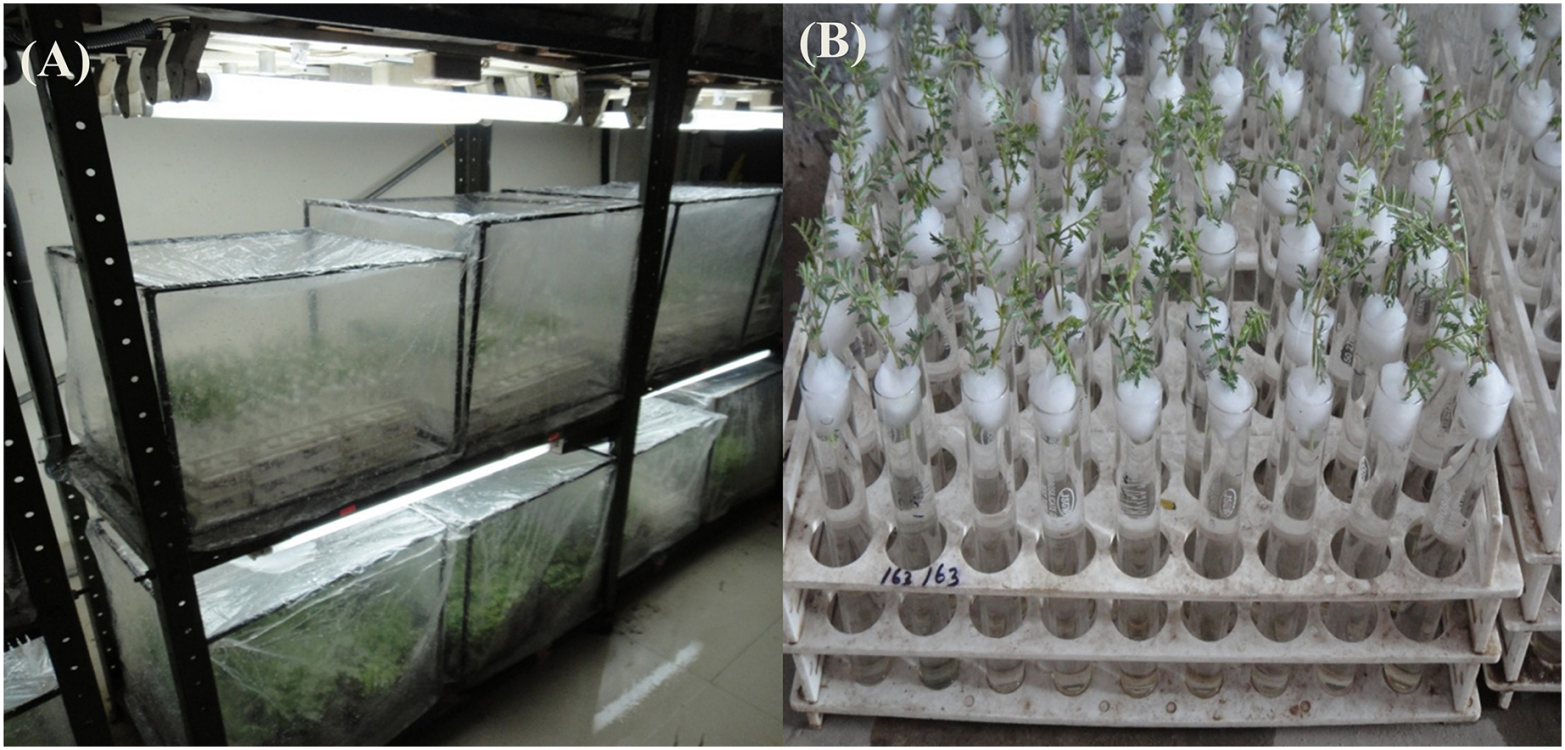
(A) Chickpea genotypes (*C. arietinum*PBG5 and *C. pinnatifidum*188) kept in growth chambers (inoculated and control). (B) Cut twigs of *C. pinnatifidum*188.

The material comprised an annual accession of wild *Cicer* (*C. pinnatifidum*188), already identified for possessing resistance against BGM using standard screening techniques (Gurha, Singh & Sharma, 2003), and an indigenous chickpea genotype *C. arietinum*PBG5, susceptible to *B. cinerea*. The material was procured from the Pulses section, Department of Plant Breeding and Genetics, Punjab Agricultural University, Ludhiana. Ten seeds of each genotype were undertaken for the study. Polyethylene pots of size 15 cm × 10 cm were filled with sandy-loam soil under glasshouse conditions, and 10 seeds of the PBG5 genotype were sown in each pot. After 1 month, the plants were transferred to chambers in the growth room, watered, and inoculated with a spore suspension of *B. cinerea* (1 × 10^4^ spore mL^−1^) (Kaur et al., 2013). These plants were kept in moist chambers for 1, 3, 5, and 7 days separately with 16 h light and 8 h dark periods provided through a fluorescent lamp (24" × 1.5", W 20, 32 m/W.) as depicted Figs. 1A and 1B.

### Inoculum preparation and disease survey

**Figure 2:**
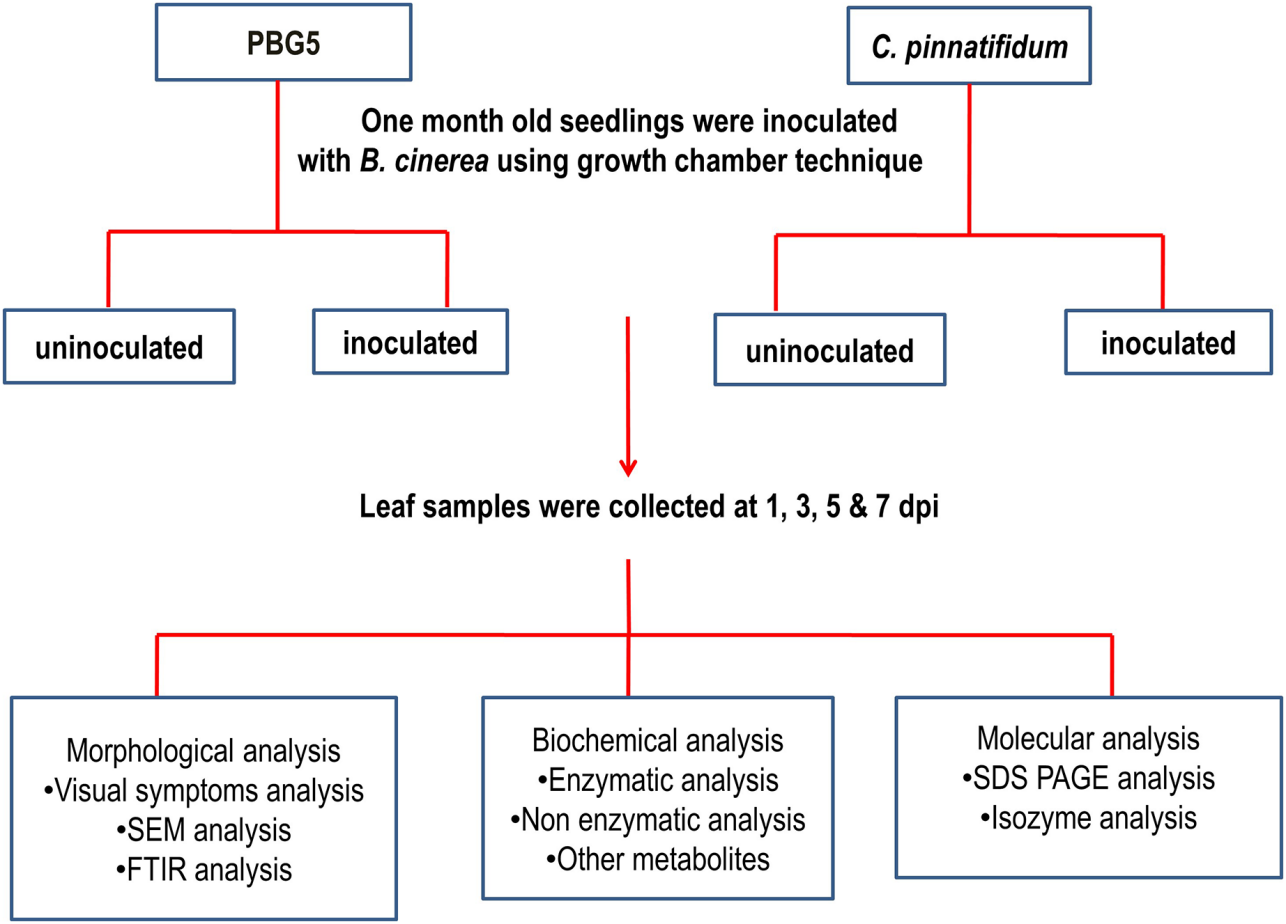
Representation of overall methodology used in the study.

Inoculum preparation, isolate 24, and race 510 of *B. cinerea* were used (Singh & Bhan, 1986). The final concentration of 1 × 10^4^ mL^−1^ spore of fungal suspension was used to inoculate both genotypes. Chickpea leaf samples were collected from both inoculated and uninoculated plants (control) on the 1^st^, 3^rd^, 5^th^, and 7^th^ days after inoculation for subsequent biochemical and microscopic (SEM and FTIR) analyses. These samples were processed immediately for enzyme extractions. The disease development was assessed by disease incidences in terms of visual leaf damage and recorded on a 1–9 point rating scale (Gurha, Singh & Sharma, 2003). Three replications each were used for enzymatic analysis. The graphical representation of the overall methodology is described in Fig. 2.

### Enzyme extractions and assays

The quantification of antioxidant enzymes was carried out spectrophotometrically using fresh leaf samples obtained from both inoculated and control chickpea genotypes. For APX and CAT, leaf tissue was extracted in prechilled 50 mM sodium phosphate buffer (pH 7.5), containing 1% polyvinylpyrrolidone (PVP) in a pestle mortar. The homogenate was centrifuged at 10,000 ‘g’ for 15 min at 4 °C and obtained supernatant was used for the spectrophotometric assays of (APX, EC 1.11.1.11), (CAT, EC 1.11.1.6), (SOD, EC 1.15.1.1),(POD, EC 1.11.1.7), (PPO, EC1.10.3.2), and (PAL, EC4.3. 1.24).

APX activity was determined as described by Yang et al. (2008). The activity was recorded as a decrease in absorbance at 290 nm upto 3 min at 25 °C at the interval of 30 s and expressed as µmole of ascorbate decomposed min^−1^ g^−1^ fresh weight of tissue using 2.8 mM^−1^ cm^−1^ as extinction coefficient of ascorbic acid. The method (Aebi, 1984) recorded the catalase activity at 240 nm as the rate of µmole of H_2_O_2_ oxidized min^−1^ g^−1^ fresh weight using a molar extinction coefficient of 0.0394 mM^−1^ cm^−1^. SOD activity was read at 420 nm by the method of Marklund & Marklund (1974). Likewise, POD activity was determined by Chance & Maehley (1955) as a change in optical density min^−1^ g^−1^ fresh weight. The PPO assay was carried out following the method of Zauberman et al. (1991) and activity was recorded at 410 nm as the change in absorbance min^−1^ g^−1^ fresh tissue weight for 3 min at the interval of 30 s. The method calculated the PAL activity (Burrell & Rees, 1974), then measured absorbance at 290 nm.

### Non-enzymatic antioxidants and other related metabolites

#### Extraction and assay of non-enzymatic antioxidants

H_2_O_2_ estimation was done as described by the method of Alexieva et al. (2001), and the absorbance was read at 390 nm. Proline content was evaluated by the procedure described by Bates, Waldren & Teare (1973). For AA determination, tissue was extracted, followed by the method of Nobuhiko et al. (1981), and absorbance was measured at 525 nm. For GSH, the sample was homogenized in 5 ml of Tris HCl (pH 7.0) and estimated by Habig, Pabst & Jakoby (1974).

#### Extraction and estimation of other related metabolites

Total phenolic content was estimated according to the method of Swain & Hillis (1959) and absorbance was read at 760 nm. To measure the levels of oxidative damage in both *B. cinerea*-inoculated and uninoculated leaf samples, the MDA concentration (a byproduct of lipid peroxidation) was quantified using the method outlined by Heath & Packer (1968). The absorption was recorded at 532 nm and nonspecific assimilation at 600 nm was subtracted from absorbance at 532 nm. The MDA content was calculated using its molar extinction coefficient of 155 mM^−1^ cm^-1^ and expressed as mole g^−1^ fresh wt.

### Isoenzymic analysis and activity staining of enzymes

Isozymic analysis of various enzymes was done at various stages in control and inoculated chickpea leaves. Electrophoretic analysis was carried out in 9% resolving gel for CAT, SOD and POX, APX and PPO 7% resolving and 4% stacking gel was used with a persistent electric current of 50 mA at 4 °C. Non-denaturing conditions were maintained in tris-glycine buffer (25 mM tris and 192 mM glycine, pH 8.3) using a Genei Mini protein electrophoretic system. An equal amount of protein (enzyme extract) from each sample was loaded on the gels.

#### Extraction and staining of antioxidant enzymes

For CAT and SOD analysis, the Extraction was done using the protocol of Medici et al. (2004). For POD and PPO analysis, the Extraction was performed in 50 mM sodium phosphate buffer (pH 7.0) (Reuveni, 1995). The extract thus obtained was used for staining assays. Protein content in these extracts was measured by the method of Lowry et al. (1951) using BSA as a standard. These extracts were loaded to Native PAGE for studying the isoenzyme patterns of CAT and SOD. The CAT activity was monitored using the methodology of Vitoria, Lea & Azevedo (2001).

For SOD activity, gels were immersed in potassium phosphate buffer (50 mM, pH 7.8) containing 0.05 mM riboflavin, 1 mM EDTA, 0.1 mM NBT and 0.3% TEMED for 30 min in the dark at room temperature (Azevedo et al., 1998). For POD staining, gels were immersed in 25 mM citrate-phosphate buffer (pH 5.3) containing guaiacol (5 mM) for 30 min on a shaker. H_2_O_2_ (0.01% (v/v)) was added to the gels solution, and reddish-brown bands were visualized on a transparent gel. For PPO staining, gels were washed multiple times with deionized water and placed in a sodium phosphate buffer solution (100 mM, pH 7.0), 10 mM 1,3-dihydroxyphenylalanine for 30 min on a rotary shaker. All the gels were scanned using Alpha-Imager software on a gel documentation system (ProteinSample, San Jose, USA). The relative front (R_f_) value of different isoforms of antioxidant enzymes was noted.

### Scanning electron microscopic (SEM) analysis of leaf samples

Control and fungal inoculated chickpea leaves were processed for SEM analysis according to the method described (Alves & Pozza, 2012). Leaves were cut into small segments with a razor blade in thin layers of less than 0.5 to 1 cm. The specimens were pasted using adhesive tapes on the surface of stubs covered with aluminium and submitted to the metallization with gold using ion sputter coater (Hitachi model E-1010) equipment and photographed by scanning electron microscope (Model Hitachi S-3400N, Hitachi Co. Ltd, Tokyo, Japan).

### Fourier transform infrared (FTIR) analysis of leaf samples

#### Protein extraction and spectroscopic analysis

One gram leaf sample was extracted with 25 mM Tris HCl buffer (pH 8.3). Then the extracts were used for FTIR analysis. One drop of each protein extract was poured into the pellet, forming disc. Pressed the sample at 10,000 psi with a hydraulic press and placed it in an FTIR sample holder. Spectra were recorded in the frequency region 450–4,000 cm^−1^ under a resolution of 0.1–0.3 cm^−1^ and with a scanning speed of 0.05–5 cm sec^−1^ on a Thermo Fisher Scientific Spectrum RX-IFTIR spectrophotometer (Thermo Fisher Scientific, Waltham, MA, USA).

### Statistical analysis

All biochemical parameters were analyzed in triplicate employing ANOVA (complete random design) at a 5% significance level using the CPCS-I software of Punjab Agricultural University, Ludhiana. Values were also arranged by Tukey’s *post hoc* test using SPSS V 16.0 software to test the significant differences between mean values. Bar diagrams and tables depict the data in terms of Mean ± SD.

## Results

Enzymatic and non-enzymatic components of the antioxidant machinery are primarily responsible for maintaining redox homeostasis in plants under any stress scenario. Consequently, the objective of the present investigation was to establish a correlation between disease severity at the seedling stage and biochemical responses in diverse genotypes of chickpeas infected with *B. cinerea*.

### Visual symptoms of disease development

**Figure 3:**
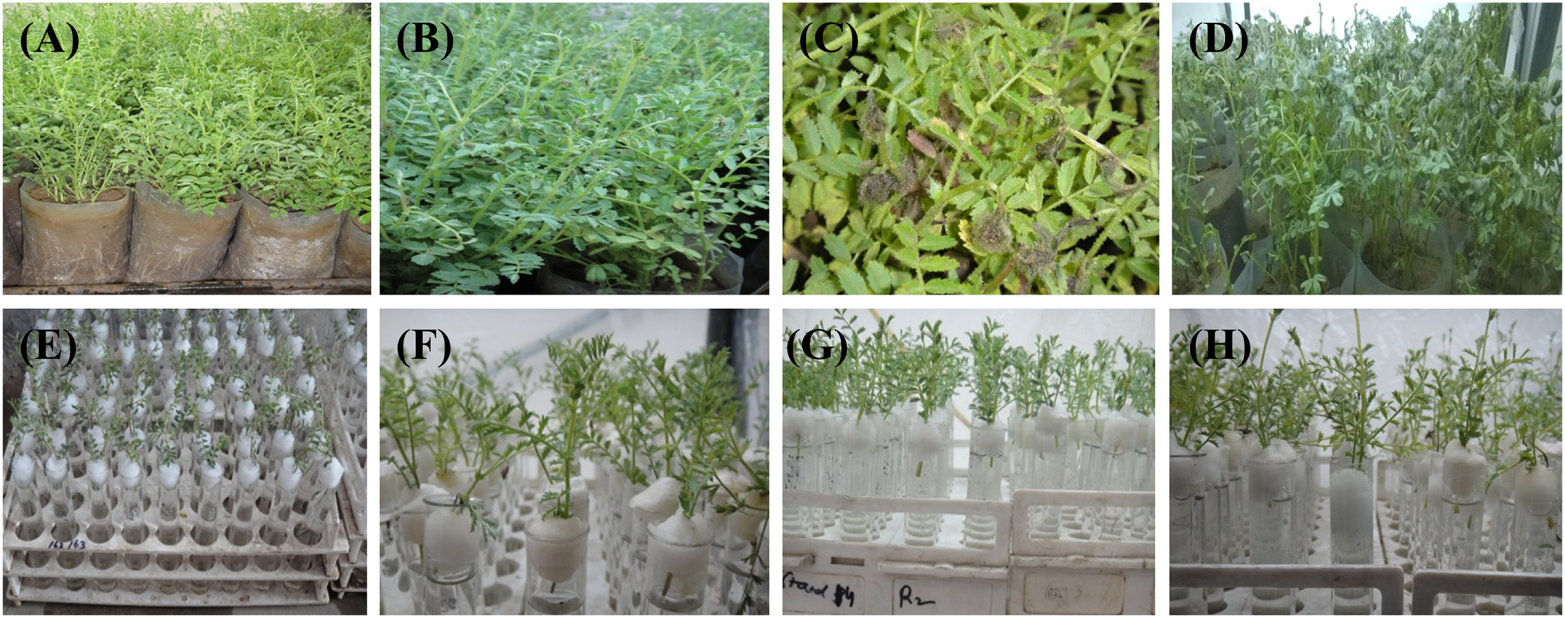
Visual symptoms of BGM infection on *C. arietinum*PBG5 (A, control; B, greyish colony on the twig; C, water soaked lesions; D, infected shoots without pods) and *C. pinnatifidum*188 (E–H) with no disease symptoms at 1st, 3rd, 5th, & 7th d.

In general, the susceptibility of various genotypes to different pathogens depends on the genetic architecture of the plant and pathogenic organism. The present study recorded disease incidences in visual leaf damage using a 1–9 point rating scale (Gurha, Singh & Sharma, 2003) (Table 1). The susceptible genotype *C. arietinum*PBG5 showed disease symptoms in water-soaked lesions, greyish colonies on the twigs and infected plants without pods on the 3^rd^, 5^th^ & 7^th^ day post-inoculation. In contrast, no such symptoms were observed in resistant genotype *C. pinnatifidum*188 at any stage after inoculation (Fig. 3). The visual observation of disease progression was further substantiated with enzymatic and non-enzymatic profiling.

**Table 1:** Disease response of leaves of PBG5 and *C. pinnatifidum* to BGM.

Scale	Symptoms + Leaf damage %			
	PBG5		*C. pinnatifidum*	
1	No infection on any part of the plant	0	No visible disease symptoms on any part	0
3	Water-soaked lesions on leaves	40	,,,,,	0
5	Rotting of leaves and tender shoots	80	,,,,,	0
7	Fungal growth on maximum leaves and shoots	>80	,,,,,	0
9	Extensive rotting	100	,,,,,	0

### Profile of antioxidant enzymes

**Figure 4:**
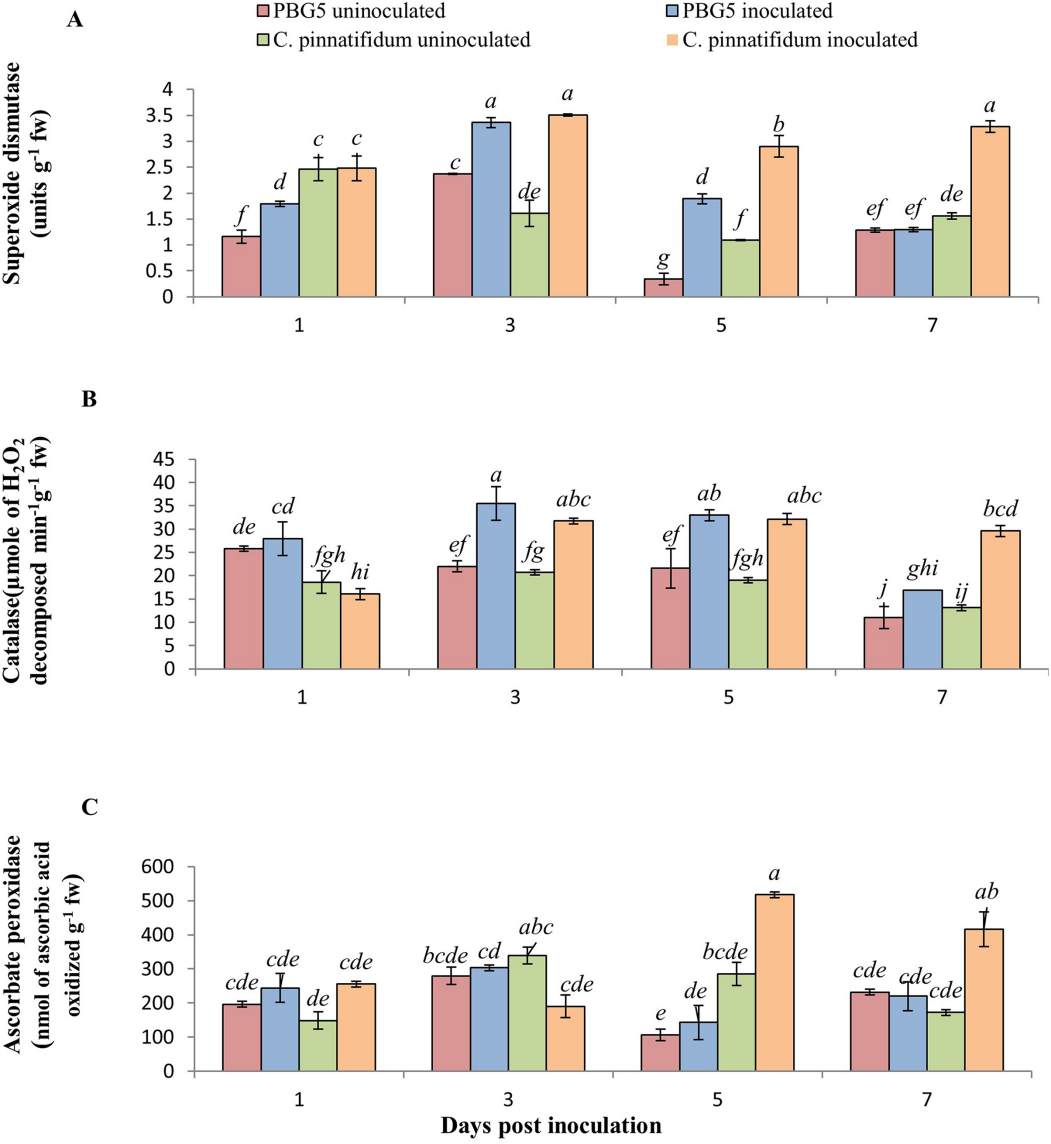
(A) Superoxide dismutase, (B) catalase, and (C) ascorbate peroxidase activities in leaves of chickpea genotype *C. arietinum*PBG5 (susceptible) and *C. pinnatifidum*188 (resistant) uninoculated and inoculated with *B. cinerea*. Data represent the mean of replications with SD as error bars. Different lowercase letters indicate significant differences among genotypes at different dpi according to Tukey’s test (*P* ≤ 0.05).

In our investigation, SOD activity in inoculated leaves of *C. pinnatifidum*188 was higher than *C. arietinum*PBG5 at all the growth stages post-inoculation. Compared to their respective controls, the activity of inoculated *C. arietinum*PBG5 and *C. pinnatifidum*188 leaves rose considerably from 1 to 5 days post-inoculation (dpi) to 3 to 7 dpi (dpi). Within 5 days post-inoculation, the activity of inoculated *C. arietinum*PBG5 leaves was 1.5 to 5 times greater than that of the control (Fig. 4A). Comparatively, the SOD activity of *C. arietinum*PBG5 and *C. pinnatifidum*188 inoculated leaves increased by 41.77 and 118.01% at 3 and 5 days post-inoculation, respectively.

CAT is a tetramer heme-containing enzyme that decomposes H_2_O_2_ into H_2_O and O_2_ (Mittler, 2017). CAT activity in control samples of both the genotypes did not vary significantly except at 1 dpi where activity was 1.5 fold higher in *C. arietinum*PBG5 than *C. pinnatifidum*188 (Fig. 4B). The CAT activity was 8.2, 61.54, 52.96 and 53.84% higher in inoculated PBG5 leaves and 15.8, 53.06, 68.89 and 118.2% in *C. pinnatifidum*188 infected plants at 1^st^, 3^rd^, 5^th^ and 7^th^ dpi, in comparison to their controls. Compared to control, earlier induction of CAT activity was observed in PBG5 at days 1 and 3, but it was delayed to 3^rd^ dpi in the resistant genotype. APX activity in *C. arietinum*PBG5 was almost similar in control and inoculated leaf samples at all stages. Higher APX activity in inoculated *C. pinnatifidum*188 leaves was recorded compared to control leaves at 5^th^ and 7^th^ dpi. APX activity was 3.5 and 2-fold higher in *C. pinnatifidum*188 leaves compared to PBG5 from 5-7 dpi (Fig. 4C).

**Figure 5:**
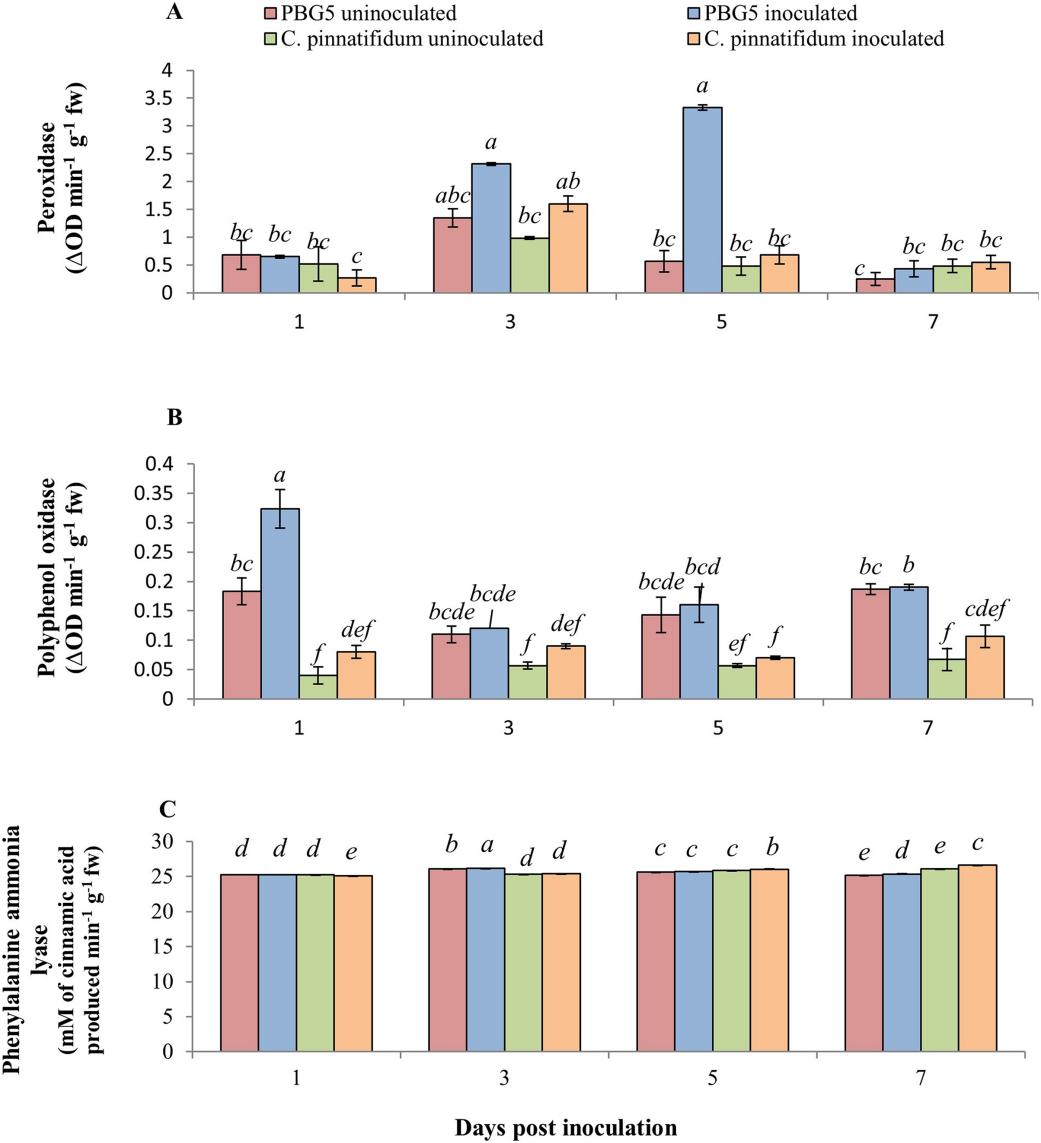
(A) Peroxidase, (B) polyphenol oxidase, and (C) phenylalanine ammonia-lyase activities in leaves of chickpea genotype PBG5 (susceptible) and *C. pinnatifidum* (resistant) uninoculated and inoculated with *B. cinerea*. Data represent the mean of replications with SD as error bars. Different lowercase letters indicate the significant differences among genotypes at different dpi according to Tukey’s test (*P* ≤ 0.05).

The inoculated and control leaf samples of *C. arietinum*PBG5 and *C. pinnatifidum*188, exhibited almost the same POD activities over the entire period. However, in inoculated *C. arietinum*PBG5 leaves, POD activity increased from 1-5 dpi and then decreased on 7^th^ day. The values were higher than control leaves from 3-7 dpi (Fig. 5A). PPO activity was significantly higher in control leaf samples of *C. arietinum*PBG5 as compared to *C. pinnatifidum*188 during the entire time course. The PPO activity increased significantly in the inoculated leaves of *C. arietinum*PBG5 at 1^st^ dpi and decreased at 3^rd^ dpi with a slight increase at 5^th^ and 7^th^ dpi, but the values were at par with that of control leaf samples. The PPO activity in inoculated *C. pinnatifidum*188 increased from 1-7 dpi compared to control plants. PPO activity was 2.5, 1.3, 3.0 and 1.8 fold higher in inoculated *C. arietinum*PBG5 leaves as compared to *C. pinnatifidum*188 at 1, 3^rd^, 5^th^ and 7^th^ dpi, respectively (Fig. 5B).

The inoculated leaves of susceptible genotype *C. arietinum*PBG5 recorded induction in PAL activity at day 1 post-BGM infection up to 7^th^ dpi compared to control leaves. In *C. pinnatifidum*188, inoculated leaves showed a decrease in PAL activity at 1^st^ dpi, then increased at 5^th^ and 7^th^ dpi compared to the control. Initially, up to 3^rd^ dpi, PAL activity was higher in inoculated leaves of PBG5. In contrast, it showed the reverse trend at 5^th^ and 7^th^ dpi *i.e*., higher activity in the resistant genotype than the susceptible one (Fig. 5C).

### Profile of non-enzymatic antioxidants and other related metabolites

#### Non-enzymatic antioxidants

**Figure 6:**
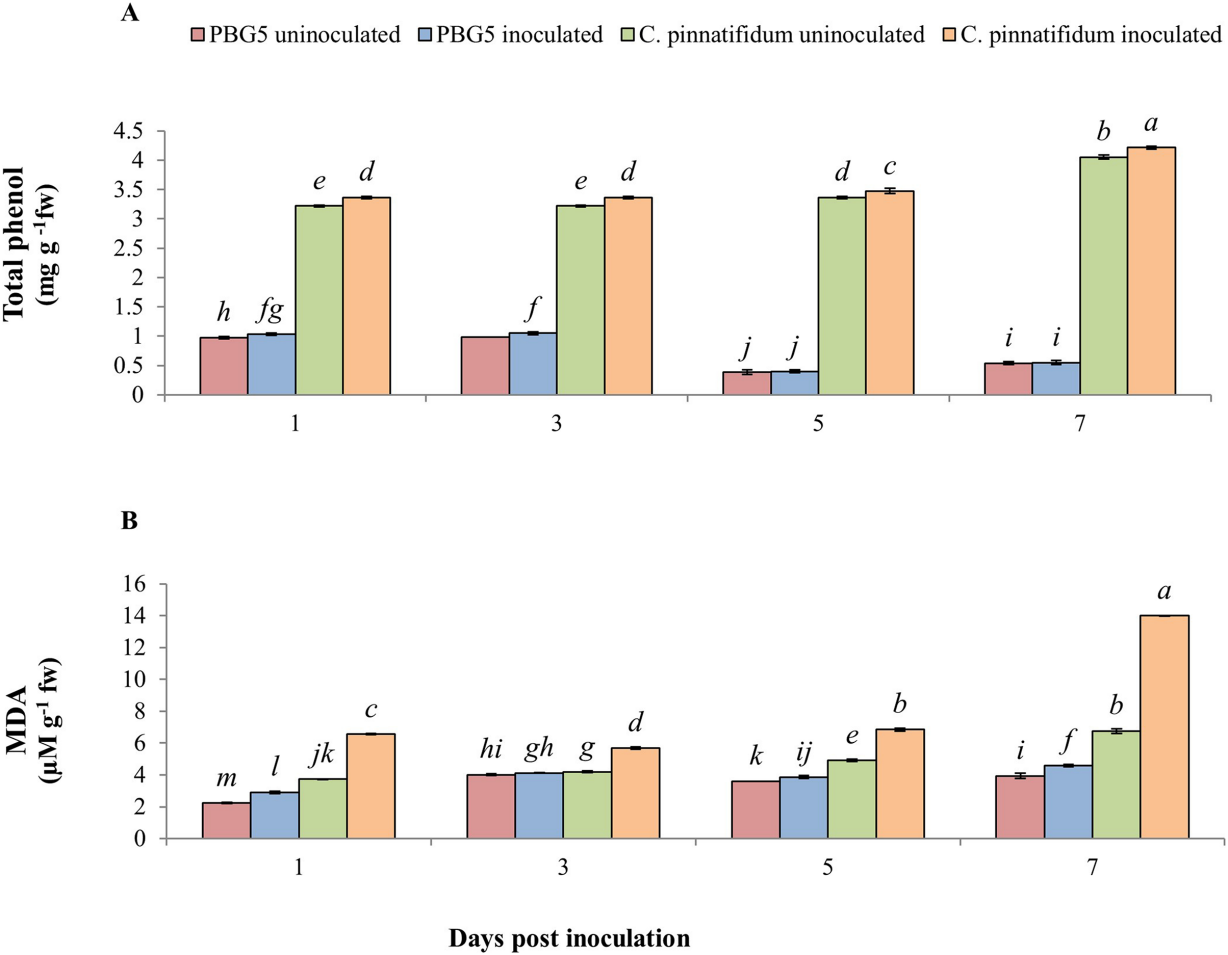
(A) H_2_O_2_, (B) proline, (C) ascorbic acid and (D) glutathione content in leaves of chickpea genotype *C. arietinum*PBG5 (susceptible) and *C. pinnatifidum*188 (resistant) uninoculated and inoculated with *B*. *cinerea*. Data represent the mean of replications with SD as error bars. Different letters indicate the significant differences among genotypes at different dpi according to Tukey’s test (*P* ≤ 0.05).

The non-enzymatic antioxidants also play a major role in the signaling and regulating host-pathogen reactions. Fig. 6 represents the effect of BGM infection on the level of various non-enzymatic antioxidants, *viz*. H_2_O_2,_ proline, GSH and AA. H_2_O_2_ production increased in both genotypes after inoculation compared to their respective controls. Higher H_2_O_2_ levels were observed in control and inoculated leaves of *C. pinnatifidum*188 compared to *C. arietinum*PBG5 at various periods (1-7 dpi) (Fig. 6A). In *C. pinnatifidum*188, inoculated leaves showed a high level of H_2_O_2_ content with a significant increase at the 5^th^ dpi. Moreover, H_2_O_2_ content increased in resistant cultivars, but the percentage change was less compared to susceptible when compared to respective controls.

At 1^st^ dpi, the proline concentration of the inoculated leaves of the resistant genotype was higher than that of the susceptible genotype. Still, it declined significantly between 3 and 7 dpi, relative to the control and inoculated samples of the susceptible genotype (Fig. 6B). In inoculated leaves of *C. arietinum*PBG5, high proline content was reported at the 5^th^ dpi; followed by similar trends at the 3^rd^ and 7^th^ dpi compared to control samples. Inoculated leaves of *C. pinnatifidum*188 showed higher proline content than control leaves during the entire time course. GSH is known to be a potent intracellular defensive metabolite capable of quenching toxic ROS (Singla et al., 2020a). It also participates in ascorbic acid regeneration and is used as a stress marker (Pandey et al., 2015). At 1^st^ dpi, GSH levels were higher in *C. arietinum*PBG5 compared to *C. pinnatifidum*188 and its production increased from 3-7 dpi in both the genotypes. After inoculation, GSH levels increased significantly in leaves of both the genotypes with an almost 1.5-fold higher increase in *C. pinnatifidum*188 as compared to *C. arietinum*PBG5 (Fig. 6C). AA is the most abundant, potent, and water-soluble antioxidant that minimizes plant damage caused by ROS (Akram, Shafiq & Ashraf, 2017). AA content increased significantly in inoculated leaves of both the genotypes with a maximum increase in *C. arietinum*PBG5. Both genotypes showed a decreasing trend in AA levels in inoculated leaves from 3-7 dpi. *C. arietinum*PBG5 control leaves showed low AA content at 1 & 3^rd^ dpi and followed an upward trend up to 7^th^ dpi compared to *C. pinnatifidum*188 (Fig. 6D).

#### Other related metabolites

**Figure 7:**
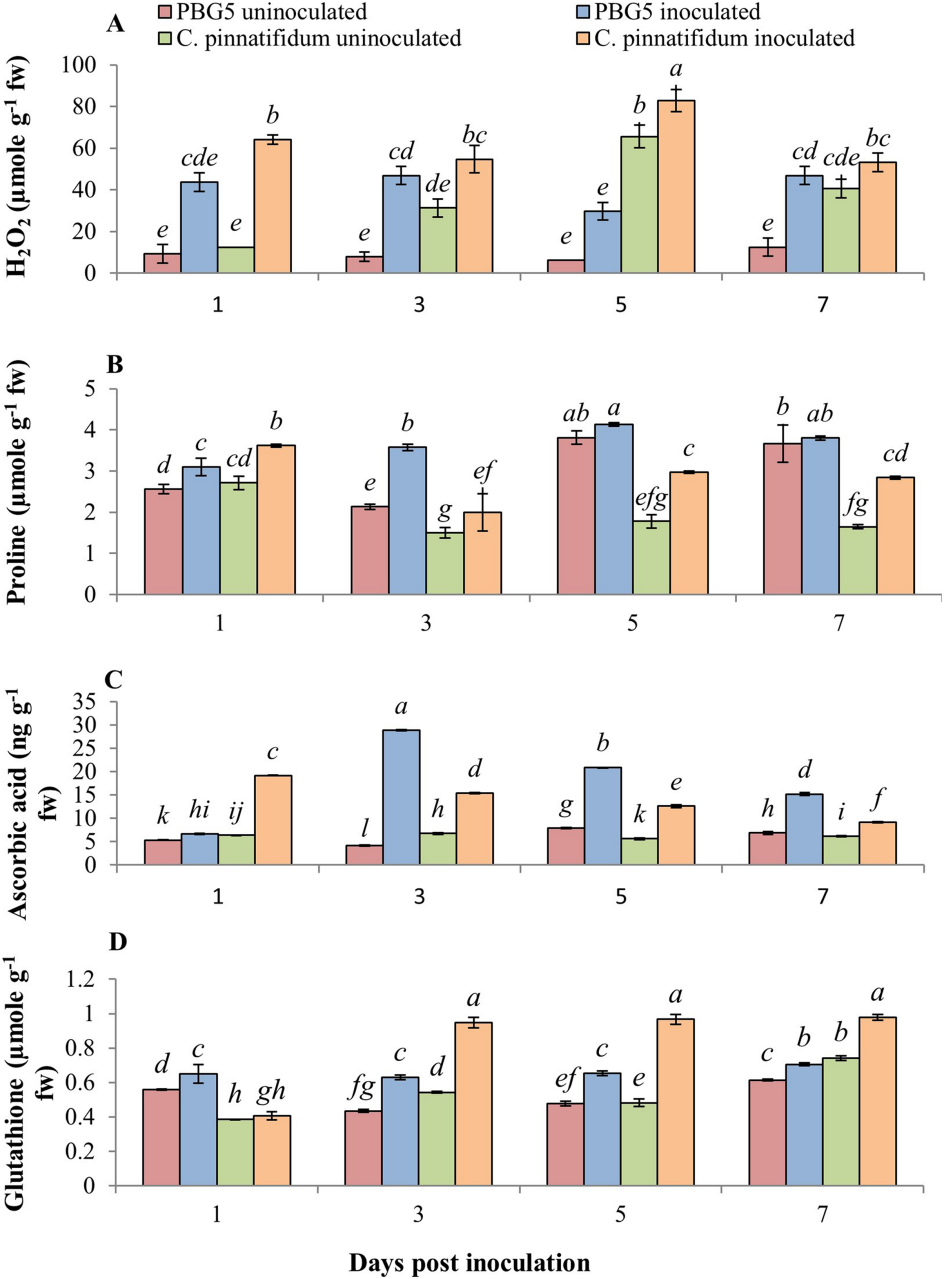
(A) Total phenol and (B) MDA content in leaves of chickpea genotype *C. arietinum*PBG5 (susceptible) and *C. pinnatifidum*188 (resistant) uninoculated and inoculated with *B. cinerea*. Data represent the mean of replications with SD as error bars. Different lowercase letters indicate significant differences among genotypes at different dpi according to Tukey’s test (*P* ≤ 0.05).

In our experiment, total phenolic content (TPC) was significantly higher in inoculated and control samples of *C. pinnatifidum*188 leaves as compared to *C. arietinum*PBG5 from 1-7 dpi. TPC increased in inoculated *C. arietinum*PBG5 leaves up to 3^rd^ dpi and decreased until the 7^th^ dpi. TPC increased significantly in inoculated *C. pinnatifidum*188 leaves compared to control plants from 1-7 dpi. TPC was about three fold higher in both uninoculated and inoculated leaves of *C. pinnatifidum*188 up to 3 days and then showed a 7-8 fold increase from 5^th^ to 7^th^ dpi in comparison to *C. arietinum*PBG5 (susceptible) genotype (Fig. 7A). Control leaf samples of *C. pinnatifidum*188 showed significantly higher MDA levels as compared to *C. arietinum*PBG5 leaves during the entire growth period. MDA levels significantly increased from 1-7 dpi in inoculated leaves of both genotypes compared to respective controls. Mean MDA content was almost two fold higher in inoculated leaves of resistant genotype than uninoculated ones. Changes in MDA contents in both uninoculated and inoculated leaves of both genotypes showed an almost similar pattern during crop growth (Fig. 7B).

### Isozymic analysis of antioxidant enzymes

**Figure 8:**
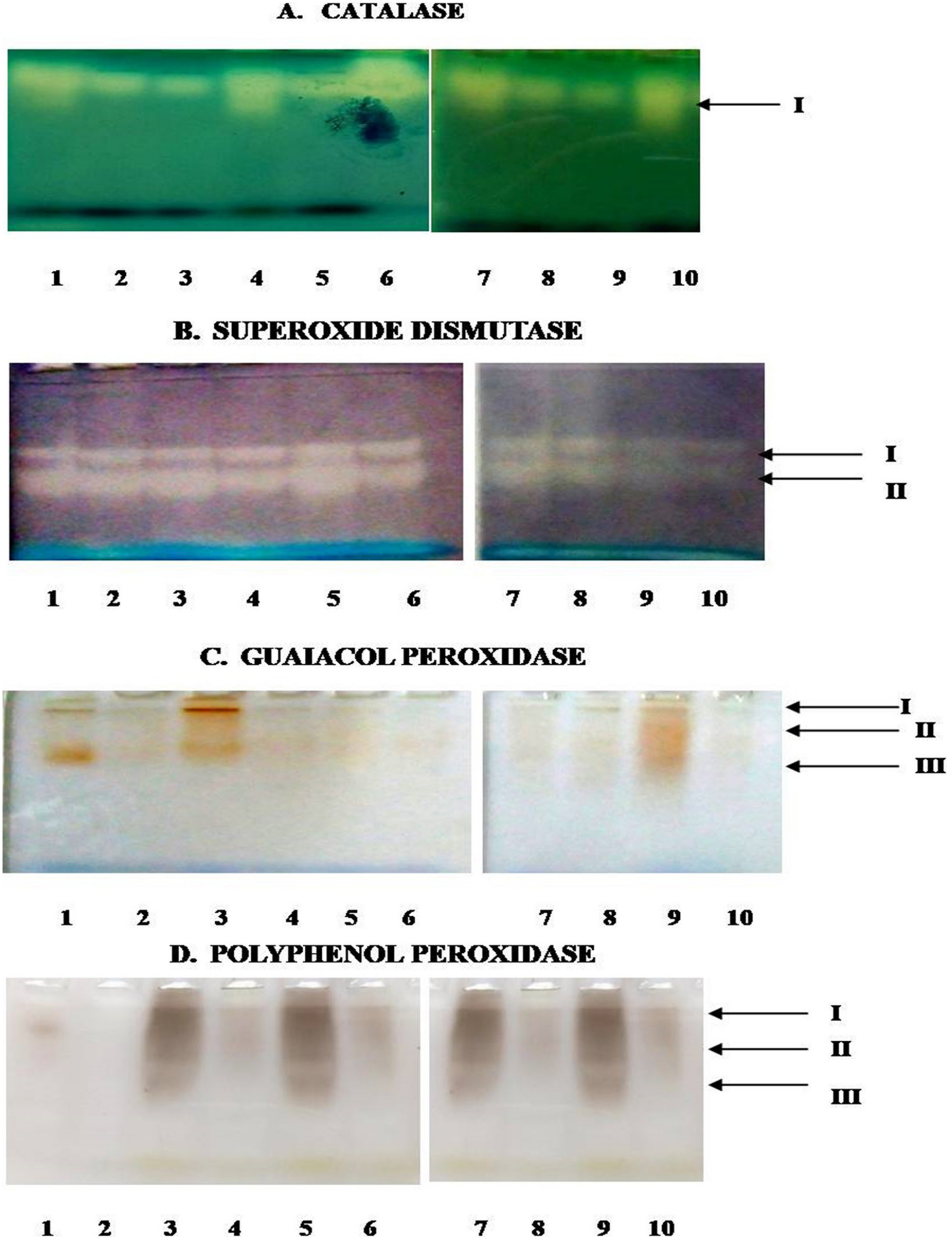
Activity staining of different antioxidant enzymes following native PAGE of leaf extracts of susceptible and resistant chickpea genotypes. (A) Catalase. (B) Superoxide dismutase. (C) Guaiacol peroxidase. (D) Polyphenol peroxidase. 1–10—represent no. of wells, arrows indicate—no. of bands, 1—*C. arietinum*PBG5 control, 2—*C. pinnatifidum*188 control, 3—*C. arietinum*PBG5 (day 1), 4—*C. pinnatifidum*188 (day 1), 5—*C. arietinum*PBG5 (day 3), 6—*C. pinnatifidum*188 (day 3), 7—*C. arietinum*PBG5 (day 5), 8—*C. pinnatifidum*188 (day 5), 9—*C. arietinum*PBG5 (day 7), 10—*C. pinnatifidum*188 (day 7).

In the present study, Fig. 8 depicts the pictorial representation of native PAGE analysis of isoforms of different enzymes in control and inoculated leaf samples of resistant and susceptible chickpea genotypes at 1^st^, 3^rd^, 5^th^ and 7^th^ dpi. The densitometric analysis determined the R_f_ value of different isoforms of these enzymes in inoculated and control leaf samples (Table 2). CAT showed the mono isozymic band in the gel in control and inoculated leaves of both the genotypes at all the growth stages (Fig. 8A). The intensity of the CAT band was high in control and inoculated leaves of *C. arietinum*PBG5 at 5^th^ dpi in comparison to 1^st^ & 7^th^ dpi. In *C. pinnatifidum*188, CAT isoforms showed low band intensity in inoculated leaves and band intensity increased from 1-7 dpi. These results are following the relative spectrophotometric CAT activities at different growth stages after *B. cinerea* inoculation. The non-denaturing PAGE analysis revealed two isoforms (SOD I and SOD II) in both control and inoculated leaves of both genotypes between 1-7 dpi (Fig. 8B). Interestingly, a higher intensity of SOD I was observed in *C. pinnatifidum*188, both in control and inoculated leaves from 1-5 dpi, as compared to *C. arietinum*PBG5. In contrast, isoform II showed higher band intensity in *C. arietinum*PBG5 at same sampling dates than *C. pinnatifidum*188. Fig. 8C indicates the isoform profile of peroxidase. The gel showed the presence of three isoforms of POD in control leaves of *C. arietinum*PBG5 and two isoforms in *C. pinnatifidum*188. Maximum intensity bands were observed in isoforms II and III at 1^st^ dpi in inoculated leaves of *C. arietinum*PBG5 compared to *C. pinnatifidum*188. At 3^rd^ and 7^th^ dpi, POD expressed two isoforms in *C. arietinum*PBG5 and one isoform in *C. pinnatifidum*188 inoculated leaves. Both the genotypes expressed only a single isoform with very low intensity at 5^th^ dpi.

**Table 2:** R_f_ values of different isoforms of antioxidant enzymes in uninoculated and inoculated leaves of susceptible and resistant chickpea genotypes.

Antioxidant enzymes	Isoforms	Days post inoculation									
		Control		1		3		5		7	
		S	R	S	R	S	R	S	R	S	R
Catalase	I	0.204	0.209	0.209	0.209	0.204	0.209	0.209	0.209	0.204	0.204
Superoxide dismutase	I	0.522	0.529	0.518	0.563	0.543	0.562	0.602	0.607	0.545	0.561
	II	0.669	0.657	0.547	0.718	0.569	0.543	0.714	0.729	0.622	0.575
Guaiacol peroxidase	I	0.071	0.108	–	–	–	–	–	–	–	–
	II	0.160	0.122	0.187	0.095	–	0.095	0.095	0.111	0.054	–
	III	0.423	–	0.204	0.104	0.123	–	0.290	0.323	–	–
Polyphenol peroxidase	I	–	–	0.065	–	0.065	–	0.063	–	0.063	–
	II	0.294	–	0.134	0.156	0.134	0.159	0.132	0.159	0.133	0.156
	III	–	–	0.381	–	0.373	–	0.377	–	0.374	–

**Note:**

-, not detected; S, susceptible genotype (PBG5); R, resistant genotype (*C. pinnatifidum*).

The expression of PPO Isozyme activity on the gel is presented in (Fig. 7D). Uninoculated leaves of both the genotypes did not show PPO activity in gel except for a very low-intensity band corresponding to isoform II in *C. arietinum*PBG5. After inoculation, *C. arietinum*PBG5 expressed three isoforms (I; R_f_ 0.063–0.065, II; R_f_ 0.132–0.156, III; R_f_ 0.370–0.381) at 1-7 dpi in comparison to *C. pinnatifidum*188 which showed only one isoform (PPO II).

### SEM analysis

**Figure 9:**
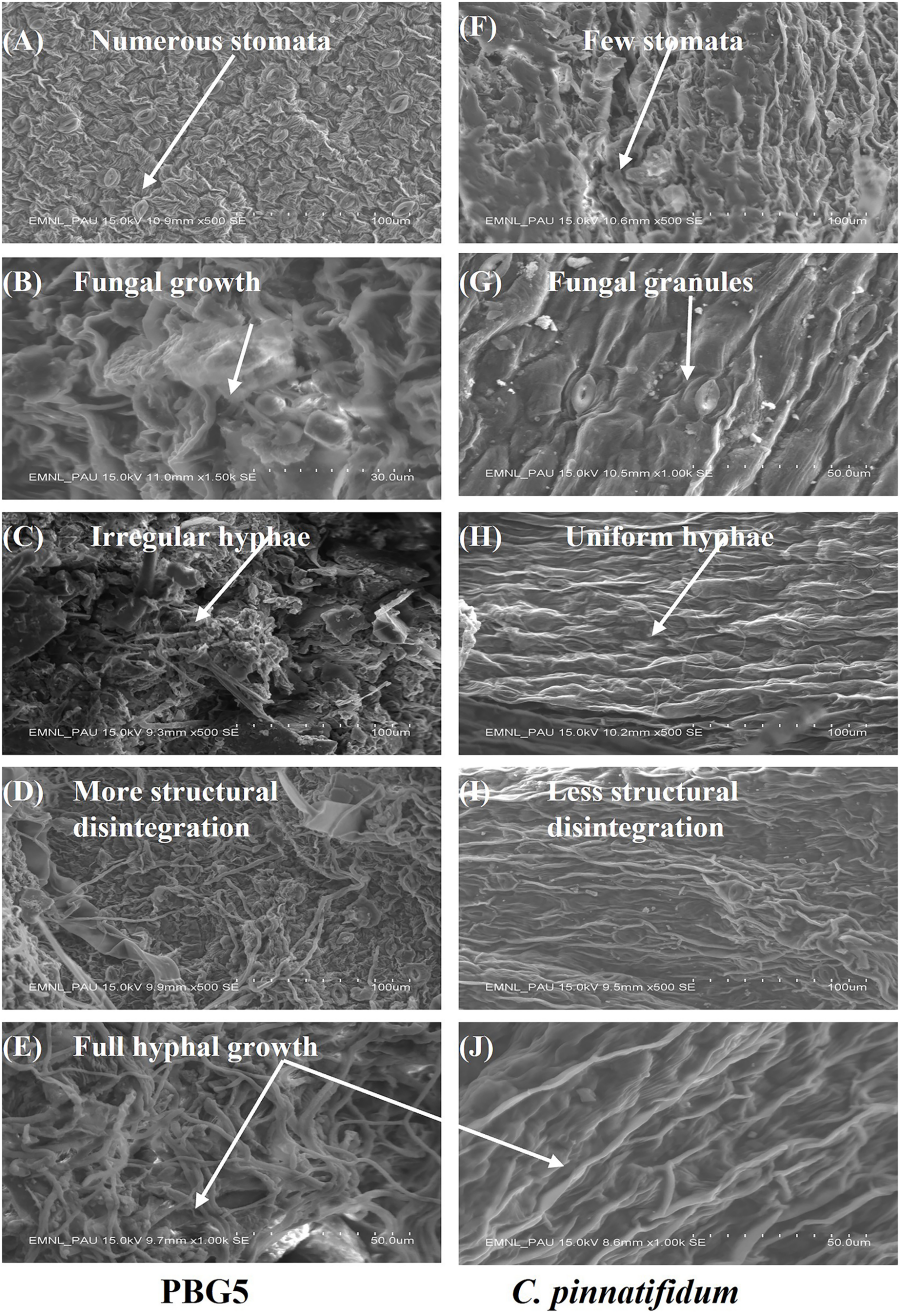
SEM analysis of uninoculated and *B. cinerea* inoculated leaf samples of susceptible and resistant chickpea genotypes: (A) control *C. arietinum*PBG5, (F) control *C. pinnatifidum*188) showing variation in no. of stomata, (B) fungal growth seen in *C. arietinum*PBG5, (G) fungal granules seen in *C. pinnatifidum*188) at day 1, (C) irregular and rough hyphal network in *C. arietinum*PBG5, H-uniform and smooth hyphal network in *C. pinnatifidum*188) at day 3, (D) more cell surface topographical disintegration in *C. arietinum*PBG5, (I) less topographical disintegration in *C. pinnatifidum*188) at day 5, (E & J) full hyphal network developed in *C. arietinum*PBG5 & *C. pinnatifidum*188) at day 7.

SEM analysis was done at different resolutions to examine the ultrastructural changes in the control and BGM-infected leaf samples of susceptible and resistant chickpea genotypes (Fig. 9). In the control samples of both the genotypes, leaf surface topography was observed to be different and the number of stomata also varied (Figs. 9A and 9F). The control leaf samples of *C. arietinum*PBG5 exhibited higher numbers of stomata than *C. pinnatifidum*188. Leaf samples of *C. arietinum*PBG5, showed a more prominent fungal growth with partial disintegration of the cell surface, while in *C. pinnatifidum*188, small fungal granules were observed without any damage to surface topography (Figs. 9B and 9G). At 3^rd^ dpi, more disintegration of the surface was observed due to fungal invasion in *C. arietinum*PBG5, while minor hyphal growth was seen on the surface of *C. pinnatifidum*188 (Figs. 9C and 9H). Moreover, the surface structure of leaf samples of *C. arietinum*PBG5 was found to be rugged and irregular, while in the case of *C. pinnatifidum*188, a smooth and uniform surface texture was observed. With the increase of fungal infection, hyphal growth also increased at day 5 in both susceptible and resistant chickpea genotypes (Figs. 9D and 9I). Finally, at 7^th^ dpi, the entire leaf surface was fully embellished by fungal hyphae, which were irregular in the case of *C. arietinum*PBG5 and uniform in *C. pinnatifidum*188 (Figs. 9E and 9J).

### FTIR analysis

**Figure 10:**
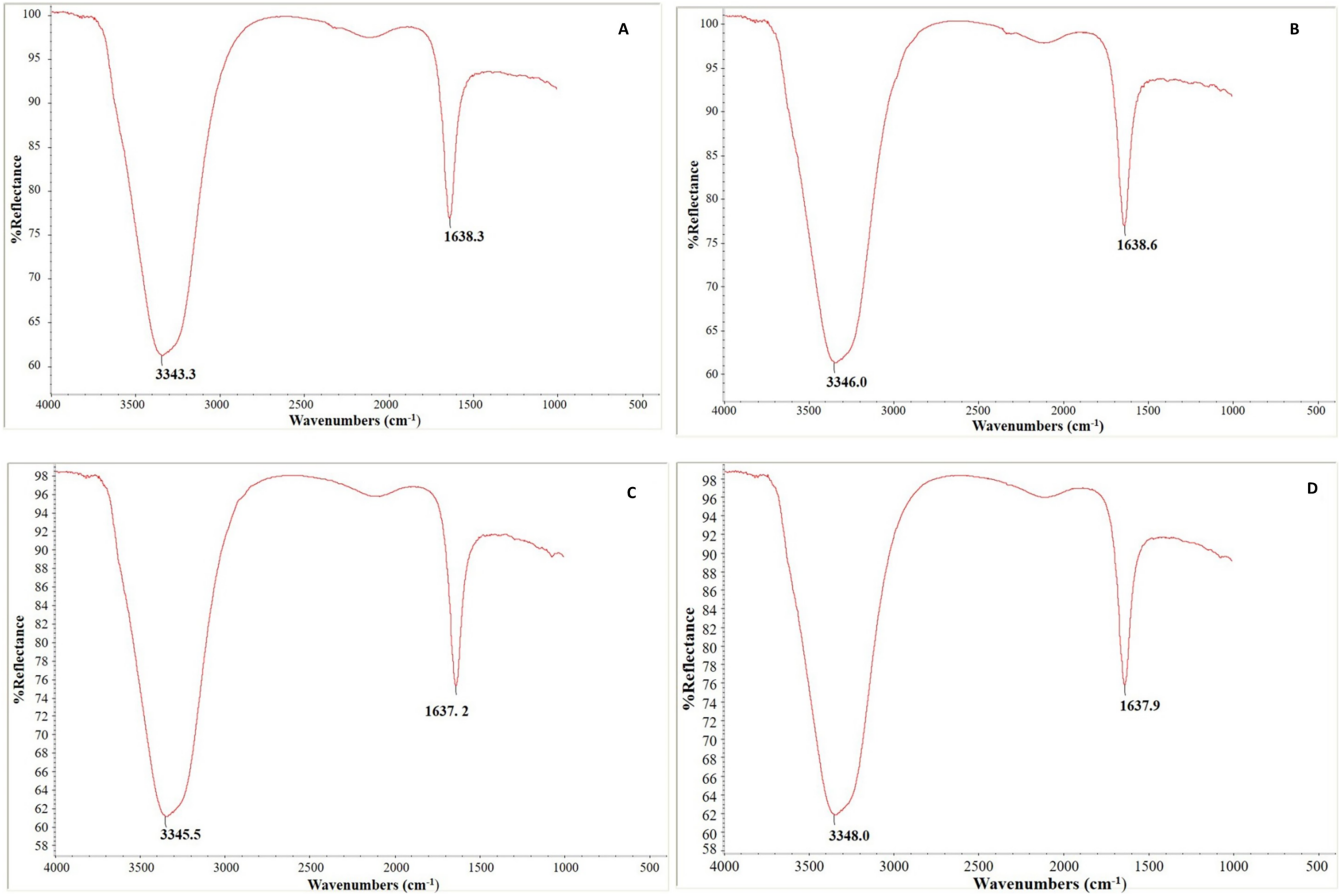
Fourier transform infrared spectroscopy analysis: (A & B) uninoculated and inoculated *C. arietinum*PBG5 and (C & D) uninoculated and inoculated *C. pinnatifidum*188 leaf samples.

In our experiment, Fig. 10 depicts each genotype’s FTIR absorption spectra of control and infected leaf samples. The leaf samples of both genotypes had two notable peaks, a broad one in the region of 3,343–3,347 cm^−1^ that may correlate to the typical polymeric OH stretch and a second (narrow) peak in the range of 1,637–1,638 cm^−1^ that corresponds to amide II. Absorption peaks were identified at 3,343.3 and 1,638.3 cm^−1^ in control leaf samples and at 3,346.0 and 1,638.6 cm^−1^ in *C. arietinum*PBG5 infected leaf samples (Figs. 10A and 10B). Similarly in *C. pinnatifidum*188, peaks were observed at 3,345.5 and 1,637.2 cm^−1^, in control and at 3,348.0 and 1,637.9 cm^−1^ in inoculated leaf samples, respectively (Figs. 10C and 10D).

## Discussion

During the activation of a plant’s defense mechanism in response to any stress, morphological changes, intensified activities of enzymatic and non-enzymatic enzymes, and other associated metabolites are primarily recognized. Under identical development conditions and inoculation duration, the pattern of visual illness symptoms varied between genotypes. These findings agreed with the studies of El Rahman et al. (2012), in which two isolates of *B. cinerea* namely B8403 and B191, infected the leaves of tomato plants resulting in lesion expansion at 4 dpi in the case of B191 but not in B8403 isolate. B191 isolate induced highly necrotic lesions with high disease intensity compared to B8403. Similar reports have been found by McGrann & Brown (2018), who observed initial small brown pepper spots on the leaves of *Ramularia collocygni* inoculated plants of different barley cultivars after 8-10 dpi. Comparatively, a vulnerable cultivar (Braemar) transmitted the illness more rapidly than a partially resistant cultivar (Power). As the disease progressed, these spots became huge lesions, finally causing the susceptible cultivar to perish. These findings validate our results and show that a highly developed immune system in a resistant genotype may resist the illness signs that arose in susceptible genotypes following infection (El Rahman et al., 2012; McGrann & Brown, 2018).

The induced activity of various antioxidant enzymes during fungal infection provides valuable insights into the activation of the plant defense system. Numerous studies have reported the critical role of enzymes such as SOD, CAT, APX, PPO, and PAL in reducing the damage caused by pathogen infection to plant cells (Fortunato et al., 2015; Gharbi et al., 2016). Our study found that the resistant genotype demonstrated higher activity levels of SOD and CAT enzymes in response to BGM. This suggests that these enzymes effectively control the toxic effects of ROS caused by the pathogen. Our results are consistent with the activation of the plant’s defence system, as evidenced by the increased activity of SOD and CAT enzymes observed following *Corynespora cassiicola* infection in soybean leaves (Fortunato et al., 2015).

Similarly, when infected with BGM, resistant genotypes exhibited greater APX activity than susceptible genotypes. This overproduction of APX can boost POD activity, strengthening the ROS-scavenging mechanism and resisting oxidative stress (Yalcinkaya et al., 2019). Thus, increased ROS toxicity may be less detrimental to resistant genotypes than susceptible ones. The results of the present study were supported by various plant microbial interactions viz. soybean/*C. cassiicola* (Fortunato et al., 2015), Olive/*Verticillium dahlia* (Gharbi et al., 2016) and Chickpea/*Fusarium wilt* (Patel et al., 2017) in both resistant and susceptible genotypes.

In this study, higher POD and PPO activities in resistant cultivar correlate to the synthesis and accumulation of phenolics in chickpea leaves after *B. cinerea* infection. Polyphenolic compounds may lead to ROS reduction indirectly by triggering the activation of various antioxidant enzymes such as SOD, CAT, and APX. Activation of these enzymes after the fungal attack is an important mechanism of plants against pathogens by accumulating toxic quinines (War et al., 2012). POD activity in the present study follows the findings of Anushree et al. (2016) in rice/*Magnaporthe oryzae* interactions. Various studies have demonstrated the PPO enzyme’s role in plants’ defense mechanism against pathogen attack (Nikraftar et al., 2013; War et al., 2012). A significant increase in PPO activity was reported in plant-pathogenic fungal interaction of chickpea/*Fusarium oxysporum* (Patel et al., 2017) in resistant and susceptible than the un-inoculated controls. PAL activity changes in response to different abiotic (wounding, drought, salinity, heavy metals) and biotic (infection by fungi, bacteria, or viruses) stress in plant cells (MacDonald & D’Cunha, 2007; Gao et al., 2008). The increase in PAL activity after *B. cinerea* inoculation in chickpea genotypes in the present study might be related to the plant’s defence response against the pathogen. Anushree et al. (2016) reported increased PAL activity in resistant and susceptible rice genotypes infected with *M. oryzae*. Activity in the resistant cultivar was 63.5% higher than in the susceptible cultivar.

The non-enzymatic antioxidants also play a major role in the signaling and regulating host-pathogen reactions. H_2_O_2_ plays a remarkable role in abating plants’ biotic and abiotic stress (Huseynova, Sultanova & Aliyev, 2014). It acts as a signaling molecule at low concentrations or helps in enhanced disease resistance by increasing the expression of defence-related genes at higher concentrations, ultimately leading to programmed cell death (Smirnoff & Arnaud, 2019). In this study, BGM increased H_2_O_2_ levels in both genotypes. However higher level was found in the resistant one, thus, the resistant genotype seems to control H_2_O_2_ levels more efficiently as a low increase in H_2_O_2_ took place in the resistant one. This observed induction in H_2_O_2_ may act as a signal to accelerate the expression of defensive genes to provide resistance against disease (Huseynova, Sultanova & Aliyev, 2014).

Proline accumulation is associated with stress tolerance in various ways, and it has been observed to increase during fungal infection (Qamar, Mysore & Kumar, 2015). It acts as a potent ROS scavenger, stabilizes the structural proteins, and maintains the osmotic homeostasis in the cell, thus protecting the cell from damage due to stress (Singla et al., 2020b; Munns et al., 2019). We also reported increased proline and AA contents upon BGM infection in both genotypes. Earlier studies demonstrated the effectiveness of proline in reducing ROS under stress (Singla et al., 2020a), preventing heavy metal toxicity (Ghori, Ghori & Hayat, 2019), and delaying programmed cell death (Ghori, Ghori & Hayat, 2019; Singla et al., 2020a). Higher level of AA up to 3^rd^ dpi with further decrease up to 7^th^ dpi in inoculated *C. Pinnatifidum*188 leaves as compared to *C. arietinum*PBG5 may be due to an initial non-significant decrease in APX activity followed by higher values in resistant genotype. A strong correlation exists between phenolic constituents and resistant levels of different cultivars infected by a necrotrophic fungus (Gharbi et al., 2016). In our study, total phenols decreased in inoculated plants of susceptible genotype *C. arietinum*PBG5 while the reverse trend was observed in inoculated *C. pinnatifidum*188 plants as compared to respective un-inoculated plants. The decrease in total phenolics after inoculation in susceptible genotypes might be due to suppression of the expression of plant defence reactions, as shown by many plant pathogens during successful infection. The presence of high TPC in the wild chickpea genotype (*C. pinnatifidum*188) could be a factor in its resistance to BGM. In the present study, the MDA content of the inoculation genotypes rose relative to the control genotypes, which is consistent with Debona et al. (2012) findings. The investigations revealed a significantly higher MDA concentration in the leaves inoculated with *Pyricularia oryzae* between 2-4 dpi, as compared to the uninoculated BR18 (susceptible) and BR229 (partially resistant) wheat plants. These findings suggest that the inoculated plants experienced more oxidative damage due to the fungal infection.

Many studies have indicated the leaf enzymatic and isozyme profile alteration following pathogen infection (Hernandez et al., 2004; Melo, Shimizu & Mazzafera, 2006; Thamayanthi, Kannan & Reddy, 2007). The presence of one or more enzymatic isoforms in hypersensitive plants is connected with their increased antioxidant activity. It has been connected to resistance and the induction of pathogen-related (PR) proteins in numerous compatible and incompatible plant-pathogen interactions (Thordal-Christensen et al., 1992). The emergence of various isoforms of antioxidant enzymes may result from a change in the transcription of enzymes following fungal infection.

Many authors use SEM to get detailed ultrastructural changes in cell surfaces (Radwan et al., 2008; Pathak, Gupta & Pandey, 2012; Mondal et al., 2013). In 2015, SEM analysis of chickpea roots by Gopalakrishnan et al. (2015) observed a significant level of colonization in streptomyces-inoculated plants compared to uninoculated plants. Similarly, the ultrastructural changes were observed by Babu et al. (2018) in native and chemically treated spores of the fungus *Curvularia lunata* by SEM analysis. The observations revealed that the native preserved conidiospores displayed a smooth and uniform distribution, whereas the spores treated with chemical fixatives followed by dehydration showed distorted and extensively shrunk structures.

In our investigation, lesser and smaller stomata in resistant genotype *C. pinnatifidum*188 may be involved in conferring resistance restricting the entry of pathogen, in comparison to *C. arietinum*PBG5 with larger size and higher stomatal index that allows the entry of the pathogen and suggesting their role in susceptibility to *B. cinerea*. These observations were also validated by earlier studies which reported higher stomatal size in the susceptible watermelon genotype than in resistant one in response to Alternaria blight (Mahajan & Dhillon, 2000) and on grape genotypes against anthracnose disease (Gurjar et al., 2015). In our study, we utilized a wild Cicer species (*C. pinnatifidum*188) with a resistant genotype, which grows at a slower rate compared to the *C. arietinum*PBG5 variety. Due to the differences in growth rate and structure, we employed the cut-twig method for the resistant genotype to ensure uniformity in our experimental approach. Although the raising methods were different for the two genotypes, we used the same inoculation treatment for both by utilizing the growth chamber technique. However, it is important to note that the physiological state of a complete and healthy plant is different from that of a cut branch. This is because stress responses are triggered by the wound created during the cut twig method, which could potentially increase susceptibility to the pathogen or modify it, even when inoculated under the same conditions (Thakur et al., 2023). Therefore, while our study aims to compare the resistance of the two genotypes using standardized inoculation methods, we acknowledge the potential limitations of the cut twig method and the impact it may have on the results obtained.

In the present study, the appearance of dense mycelia growth with no mechanical disintegration in the resistant genotype suggests the possible role of hydrolytic enzymes of the pathogen on chickpea leaves. These observations were also in accordance with the studies on papaya fruit infected by *Colletotrichum gloeosporioides* (Chau & Alvarez, 1983) and Barley leaf infection by *Erysiphe graminis* (Kunoh et al., 1988). Hyphal network and roughed conidiophores of *Drechslera tetramera* were observed on wheat plants, whereas smooth-walled and slight curves of conidia of *Bipolaris orghicola* were found to be attached to the geniculate conidiophores (Gulhane, Giri & Khambalkar, 2018) by SEM analysis. These authors suggested SEM be an effective technique for observing microconidia and mycelium during fungal infections on different plants as reported earlier (El Rahman et al., 2012; Gulhane, Giri & Khambalkar, 2018).

The FTIR study of peptides and proteins is valuable because it examines the universally available amide bonds with discrete infrared (IR) signals for peptides and proteins that are folded differently (Barth & Zscherp, 2002; Adochitei & Drochioiu, 2011). It has been used to track, characterize, and research antimicrobial drugs’ modes of action (Sengupta, Mujacic & Davis, 2006; Chan et al., 2007; Szeghalmi et al., 2007). Our investigation revealed the difference in the secondary structure of the protein in terms of differences in O-H stretch and amide II peaks, in both the genotypes after fungal inoculation which may be due to the unfolding of structures with fungal inoculation. FTIR spectroscopy was also done by Withana-Gamage et al. (2011) to investigate the physicochemical, thermal and functional characterization of protein isolates from Kabuli and Desi chickpea (*Cicer arietinum* L.). The study estimated 25.6–32.7% *α*-helices, 32.5–40.4% *β*-sheets, 13.8–18.9% turns and 16.3–19.2% disordered structures by FTIR spectra. A significant increase in the intensity of nucleic acid and carbohydrate bands was observed in fungal hyphae treated with ZnO nanoparticles by FTIR spectroscopy (He et al., 2011).

Rosa-Millan et al. (2018) also performed ATR-FTIR analysis in the extracts of different pulses. The spectra showed different proportions of α helix and β sheet secondary structures of the amide II and amide III groups, reflecting inter and intramolecular associations that could have influenced their emulsion and foam characteristics. FTIR spectroscopy, Chow & Ting (2019) also detected the distinguishable influence of fungal infection on plant tissues.

## Conclusion

Food crops, especially pulses, mitigate diverse biotic stresses by adopting various survival strategies. The adaptive strategies include ROS production, antioxidant defence system activation, compatible solutes and other biochemical metabolites. Differential expressions of phenolic compounds, enzymatic and non-enzymatic antioxidants, were reported to play a significant role in resistance against pathogenic infections (Singla et al., 2020a, 2020b). In this study, various biochemical molecules such as enzymatic (SOD, CAT, PODs, PAL, *etc*.) and non-enzymatic antioxidants (H_2_O_2_, proline, AA, GSH), phenols, and MDA content were found to be elevated in both the genotypes during the interaction of *B. cinerea* with contrasting chickpea cultivars (*C. arietinum* PBG5 and *C. pinnatifidum*188). However, the resistant genotype exhibited a higher degree of elevation in these molecules than the susceptible genotype. Our study illustrated substantial changes and significant differences in the activities of biochemical molecules amidst susceptible and resistant genotypes of chickpeas during BGM infection. The results of the present study suggest the exorbitant effectiveness of the above-studied biochemical molecules in resistant chickpea genotype to combat BGM infection. SEM analysis with FTIR offers an improved understanding of fungal inoculation’s effects in diverse chickpea genotypes. Moreover, the variation in functional groups can be visualized simultaneously. Plant breeders for BGM management could exploit this information by using biotechnological techniques.

## Supplemental Information

10.7717/peerj.15560/supp-1 Supplemental Information 1 Raw data of enzymes.

## Additional Information and Declarations

### Data Availability

The following information was supplied regarding data availability: The raw data are available in the Supplemental Files.
